# Erosive Tarsal Conjunctival Lesions Following Immunogenic Events in Early Development of Ocular Graft-vs-Host Disease

**DOI:** 10.3390/life14101317

**Published:** 2024-10-17

**Authors:** Marcus G. Kohnstam, Pier Luigi Surico, Zhonghui K. Luo

**Affiliations:** 1 Massachusetts Eye and Ear, Department of Ophthalmology, Harvard Medical School, Boston, MA 02114, USA; 2 Department of Ophthalmology, Campus Bio-Medico University Hospital, 00128 Rome, Italy; 3 Department of Organs of Sense, La Sapienza University of Rome, 00185 Rome, Italy

**Keywords:** stem cell transplantation, ocular graft-versus-host disease, inflammation, conjunctival fibrosis, pseudomembrane

## Abstract

Purpose: Ocular graft-versus-host disease (oGVHD) affects more than half of the patients following allogeneic hematopoietic stem cell transplantation (HSCT). The disease onset and the pathogenesis of oGVHD are not well understood. We hope to identify the triggers and explore the clinical signs and symptoms of oGVHD development at the early stages. Methods: The records of post-HSCT patients seen consecutively in a 1-year span in a single provider’s clinic were reviewed. The history, symptoms, and clinical findings of the patients with erosive tarsal conjunctival lesions (ETCLs) were analyzed. Results: Out of the 228 patients screened, 19 had clinically witnessed ETCL in at least one eye during the period. Twelve (63%) patients had a never-before-described nodular erosion on the subtarsal conjunctiva; seven (37%) had previously described pseudomembranous erosions. The ocular symptom onset was within 1 month after immunosuppression (IS) taper, vaccination, or donor lymphocyte infusion (DLI) in 16 of the 19 patients. While 16 (84%) patients reported painless mucous discharge, only 9 (47%) reported dryness as the initial symptom. Within 6 months, only 4 (21%) had discharge but 15 (82%) patients endorsed dryness. Subepithelial conjunctival fibrosis followed ETCL immediately in situ. Corneal punctate staining increased with time, while aqueous tear production decreased. Conclusions: The ETCL described is likely one of the earliest detectable findings of oGVHD and triggered by certain immunogenic events. The ocular symptoms of wet mucous discharge should be considered a warning sign for oGVHD onset, particularly when it occurs shortly after prominently immunogenic events.

## 1. Introduction

Allogeneic hematopoietic stem cell transplantation (allo-HSCT) is an increasingly common treatment for many otherwise-fatal hematologic malignancies and disorders [1]. Chronic graft-versus-host disease (cGVHD) is the leading cause of morbidity and mortality following stem cell transplantation, affecting 30–70% of all the HSCT recipients [1,2,3].

Ocular graft-versus-host disease (oGVHD) is one of the most common presentations of cGVHD, found in about half of the patients with other types of cGVHD [4,5]. It has been shown that a donor T-cell-mediated inflammatory cascade leads to lacrimal gland fibrosis, meibomian gland dysfunction, and ocular surface inflammation, which severely affects all the components of the tear film and causes the most recognized feature of oGVHD, keratoconjunctivitis sicca (KCS) [1,6,7,8,9,10,11]. Detrimental outcomes can range widely from ocular discomfort to cicatricial deformation to corneal perforation [12,13]. Currently, treatment is mostly supportive; oGVHD can still result in debilitating pain and blindness in many patients [14].

Despite the well-recognized impact on quality of life from oGVHD [15,16], the early stages of oGVHD are not well understood. Due to the limitation of resources and the burden of diseases on patients undergoing allo-HSCT, most of them do not undergo baseline evaluation by an ophthalmologist prior to HSCT [16]. Patients often do not receive ophthalmologic care until they are severely symptomatic; therefore, mild to moderate early symptoms could go unnoticed, and the preliminary manifestations of oGVHD go undocumented and untreated. Without early recognition, early intervention is not possible, which can lead to more serious complications. It has long been recognized that early diagnosis and treatment are crucial for reducing the morbidity of oGVHD [17].

In our practice, we repeatedly found that two types of erosive tarsal conjunctival lesions (ETCLs) preceded conjunctival subepithelial fibrosis (CSEF) in situ, often before the development of keratoconjunctivitis sicca (KCS), the most recognized oGVHD clinical presentation. Although both CSEF and serosanguinous conjunctivitis have been reported before [18], there is no literature that has described a direct connection between the two. We are the first to describe the nodular type of erosive lesions on the tarsal conjunctiva, and to report the proximity in time to certain systemic immunogenic events. Our retrospective series describes what we suspect to be a critical event in the early development of oGVHD.

## 2. Materials and Methods

We conducted a retrospective observational series at Massachusetts Eye and Ear. Approval for this study (2021P003700) was obtained from the Mass General Brigham Human Research Committee of Mass General Brigham, Boston, MA, USA. All research adhered to the tenets of the Declaration of Helsinki. The electronic medical records of all the patients with a history of allo-HSCT and a diagnosis of oGVHD seen in the clinic of a single cornea specialist between 10 May 2021 and 9 May 2022 were reviewed. Among them, the patients with active erosive conjunctival lesions including nodular and/or pseudomembranous lesions involving at least one tarsus (ETCL) during at least one of the encounters were identified, and the electronic medical records of these patients were reviewed. The allo-HSCT history, immunosuppression (IS) regimen, vaccination record, and previous ocular history were summarized. The ophthalmological findings including the slit lamp examinations of the eyelids; bulbar and palpebral conjunctiva with lid eversion, cornea, and other ocular surface structures; and intraocular pressure were reviewed and analyzed. The patients underwent various treatments such as systemic immunosuppression, topical steroids (preservative-free 1% prednisolone acetate, 0.5% loteprednol etabonate suspension/ointment, or 0.05% difluprednate), punctal plugs, preservative-free artificial tears, and/or 40% autologous serum tears. Some patients required intraocular pressure control with topical agents during this period. Slit lamp photographs were obtained using Streit Haag Imaging Module 900. Anterior segment optical coherence tomography (OCT) was performed using Heidelberg Spectralis HRA + OCT. Schirmer 1 test was performed without topical anesthesia for 5 min.

## 3. Results

The electronic medical records of 228 patients with a history of allo-HSCT and a diagnosis of oGVHD were reviewed. A total of 19 patients, with a mean age of 60 years (range 29 to 72), were seen in the clinic with active ETCL involving at least one tarsus in a total of 35 eyes during the one-year period. The patients’ characteristics are shown in Table 1.

Seventeen patients presented with their ETCL at the first visit to our clinic, with or without previous ophthalmologic evaluation at other institutions following their HSCT; the other two patients presented with a new onset of ETCL with established care at our clinic following their HSCT. The 19 patients were seen in our clinic from a few days to several weeks after their symptom onset. The patients were followed in the clinic at various frequencies, and the course of ETCL was documented. In most patients, the ETCL episode was only observed once, lasting several weeks to months; in 3 eyes of 2 patients, the ETCL was witnessed to have resolved and then recurred at a later date. The diagnosis of oGVHD was either pre-existing or established after the observation of ETCL later in the course according to the NIH 2014 criteria [3].

**Figure 1:**
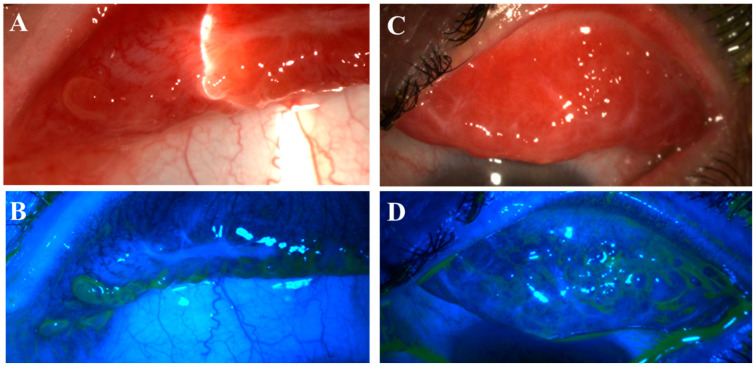
Nodular erosive tarsal conjunctival lesions (ETCLs). The slit lamp color images of everted superior palpebral conjunctiva with nodular ETCL in two representative patients following fluorescein staining without (**A**,**C**) or with (**B**,**D**) cobalt blue filter. The epithelium over the nodules displays different degrees of erosion.

**Figure 2:**
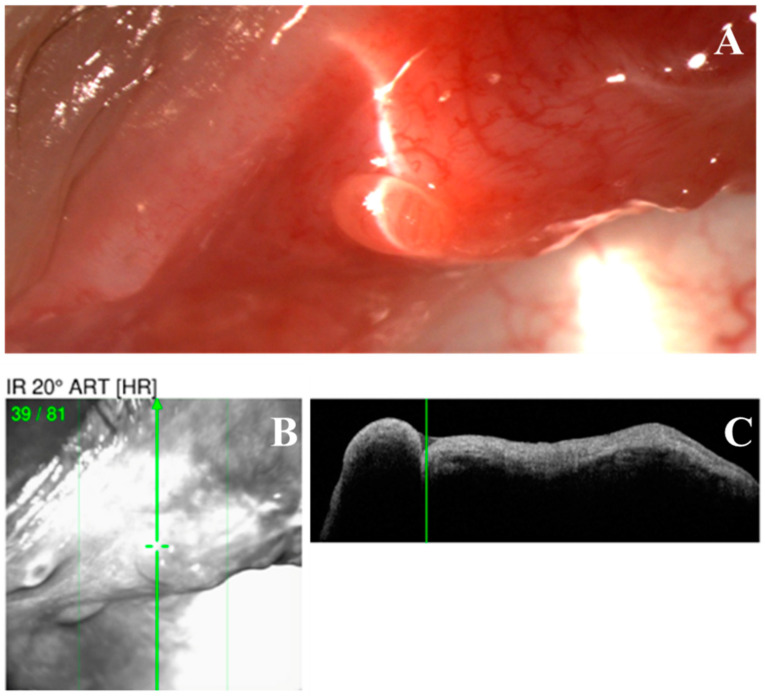
Optical coherence tomography (OCT) scan of a nodular erosive tarsal conjunctival lesion (ETCL). (**A**). The slit lamp color image of the nodule. (**B**,**C**). OCT image showing a uniformly hypo-reflective nodule without any fibrous internal structure.

**Figure 3:**
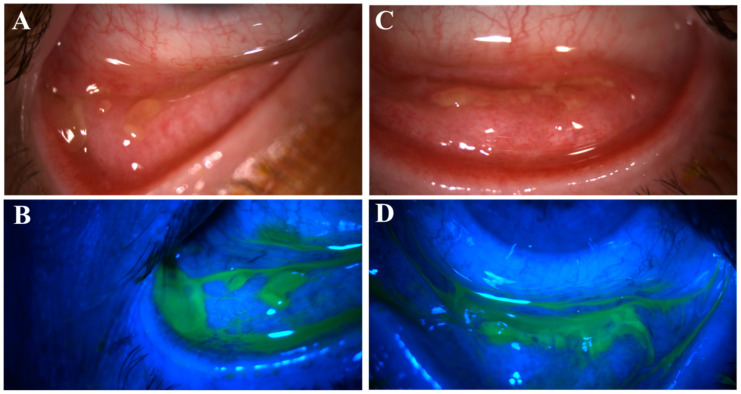
Pseudomembranous erosive tarsal conjunctival lesions (ETCLs). Slit lamp color images of inferior palpebral conjunctivae with pseudomembranous ETCL in one representative patient following fluorescein staining without (**A**,**C**) or with (**B**,**D**) cobalt blue filter.

Fifteen patients (79%) had distinctive nodular erosions (Figure 1 and Figure 2) in a total of 27 eyes; eight patients (42%) had pseudomembranous erosions (Figure 3) in a total of 14 eyes; and four patients (21%) had co-existing nodular and pseudomembranous erosions on the same tarsal conjunctiva in 6 eyes. Three eyes of 3 patients (16%) did not have any observed ETCL while the fellow eyes were actively involved.

**Figure 4:**
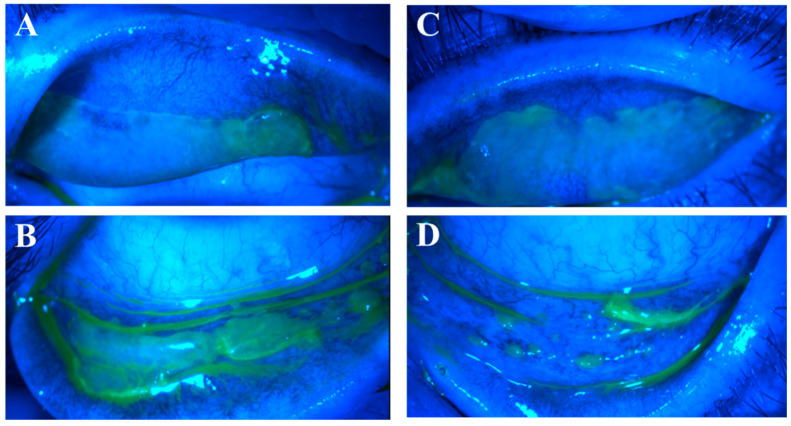
Co-existing nodular and pseudomembranous erosive tarsal conjunctival lesions (ETCLs). Slit lamp color images of all four palpebral conjunctivae with co-existing nodular and pseudomembranous ETCL in one representative patient following fluorescein staining with cobalt blue filter. (**A**). Right superior palpebral conjunctiva. (**B**). Left superior palpebral conjunctiva. (**C**). Right inferior palpebral conjunctiva. (**D**). Left inferior palpebral conjunctiva.

Of the 35 eyes with ETCL, 15 eyes had lesions (nodular and/or pseudomembranous erosions) on both superior and inferior tarsal conjunctivae (Figure 4); in the other 20 eyes, the lesions were only found on one tarsal conjunctiva, with 17 superiorly and 3 inferiorly. The superior tarsal conjunctival lesions were only visible when the upper lids were everted.

**Figure 5:**
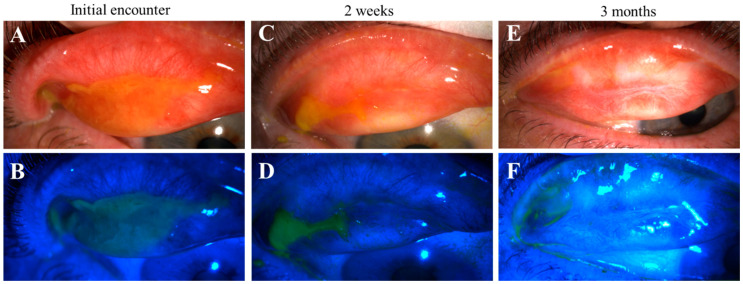
Progression of pseudomembranous erosive tarsal conjunctival lesions (ETCLs) into fibrosis. Slit lamp color images of superior palpebral conjunctivae with pseudomembranous ETCL in one representative patient following fluorescein staining without (**A**,**C**,**E**) or with (**B**,**D**,**F**) cobalt blue filter, showing progressive fibrotic changes. (**A**,**B**). At initial encounter. (**C**,**D**). Two weeks after the initial encounter. (**E**,**F**). Three months after the initial encounter.

**Figure 6:**
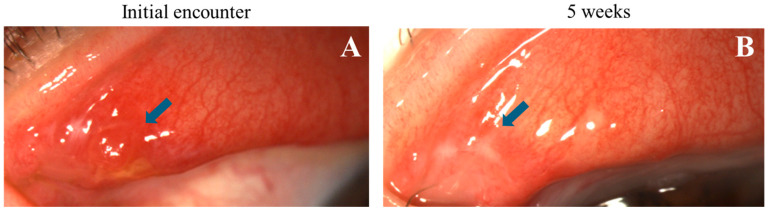
Progression of nodular erosive tarsal conjunctival lesions (ETCLs) into fibrosis. Slit lamp color images of the same superior palpebral conjunctiva (**A**). At initial encounter, a single nodular lesion (blue arrow) with adjacent pre-existing fibrosis. (**B**). Five weeks after the initial encounter, fibrosis seen at the exact location of the resolved nodule (blue arrow).

All tarsal conjunctivae of the 35 eyes following ETCL resolved and re-epithelialized with new fibrosis formation. The ETCL in 20 eyes (57%) also resulted in inferior fornix foreshortening, including inferior symblepharon formation in 3 eyes. In 16 eyes of 9 patients, both tarsal conjunctivae were free of fibrosis at the initial encounter of ETCL. In the other 19 eyes, CSEF was found adjacent to the active ETCL, and new fibrosis developed at the sites of erosions during re-epithelialization (Figure 5 and Figure 6). In the aforementioned 13 patients (23 eyes) with frequent follow-up visits, the observed ETCL completely resolved into CSEF by a mean of 2.7 months (0.5 to 6.2).

The most common initial symptoms of the 19 patients were mucous discharge or “thick tears” or “goop” (84%), morning crusting (79%), dryness (47%), and irritation/foreign body sensation (42%). Thirteen patients followed up regularly within the first 6 months after the initial presentation with ETCL. They returned to the clinic 4.6 times on average (median 4.0, range 2–9 times) within a period of 5.3 to 7.8 months after the initial encounter. The most common symptoms of these 13 patients at 6 months were irritation or foreign body sensation (69%) and dryness (77%). Concurrently, mucous discharge was only reported in 31% and crusting in 23% of the followed-up patients (Table 2).

Five patients (9 eyes) had aqueous tear production measured during the active ETCL, with the average Schirmer 1 score of 15 mm/5’ (range 0–35). Repeated tests were performed 3 to 5 months later when the conjunctiva had re-epithelialized, with the average Schirmer 1 score decreased to 4 mm/5’ (range 0–13).

The mean onset of the symptoms associated with the ETCL was 9.6 months after HSCT (range 4.2 to 21.5 months). However, the mean time between the reported symptom onset and most recent immunosuppressive drug (IS) tapering (12 patients), COVID vaccination (5 patients), post-HSCT 9-month immunization bundle (4 patients), or donor lymphocyte infusion (DLI) (1 patient) was 0.7 months (range 0.1 to 1.2). In 16 out of the 19 patients, the symptoms started within 1 month after one or more of these immunogenic events (Table 3).

A qualitative description of corneal fluorescein staining (CFS) at 5–7 months was available in these 13 patients. In seven patients, there was increased CFS in one or both eyes. Two other patients started wearing therapeutic scleral lenses after the ETCL episode; therefore, the corneal staining was non-descriptive. Three patients had decreased CFS in both eyes on an exam, and one patient never had any CFS in either eye.

All the patients had intraocular pressure (IOP) within the normal range at their initial encounters; however, three patients (16% of 19) developed an IOP greater than 21 mm Hg (mean Tmax 30.5, range 25 to 34), which required topical therapy for IOP control.

## 4. Discussion

Allo-HSCT serves as an effective cure for various hematological disorders, encompassing both malignant and non-malignant conditions. Despite significant progress in the field over the recent decades, graft-versus-host disease (GVHD) still affects more than half of the recipients and causes significant mortality and morbidity [2,19,20]. The focus of care of this population is now shifting from simple survival to quality of life of the transplantation survivors, which is significantly dependent on their ocular health. It is our hope that this retrospective series brings attention to the earlier stage of oGVHD, occurring prior to the commonly recognized, established stages when aqueous deficiency and eyelid deconfiguration have already developed from the fibrotic processes involving the ocular adnexa [18,21].

Structural changes such as conjunctival fibrosis, fornix foreshortening, and symblepharon can lead to lid retraction, entropion, ectropion, trichiasis, lagophthalmos, ankyloblepharon, and other distortions that threaten ocular surface health [22,23,24,25]. The corneal epithelium is vulnerable to exposure, particularly in the context of aqueous tear deficiency and meibomian gland disease which are both common manifestations of oGVHD [26]. Any corneal epithelial defect is more likely to lead to stromal melting, ulcer, and perforation [10]. In addition to infection, pain, and scarring, irreversible vision loss can be a very unfortunate result [25]. Furthermore, lacrimal gland fibrosis and meibomian gland damage likely happen simultaneously as the conjunctival inflammation is taking place [2,6,27,28]. Therefore, the prevention of such changes is very meaningful and only possible when the conjunctival inflammation is recognized early.

One of the most enlightening findings from our retrospective series is that during the early stage of ETCL, the most common ocular symptoms are mucous discharge and crusting, not dryness or irritation. Within 6 months, dryness becomes predominant compared to other symptoms. As oGVHD has long been recognized for and often incorrectly equated to the dry eye disease/keratoconjunctivitis sicca (KCS), the early symptoms rarely catch the attention of HSCT providers or even eyecare providers. Therefore, we should modify the expectation: the most common late manifestation of oGVHD is dryness; however, the early stage can be rather “wet”, with excessive tearing or mucous discharge. One should not rule out oGVHD simply because of a lack of dry eye complaints or findings. In addition, the onset of ETCL seems to be more associated with certain immunogenic events than the number of months after allo-HSCT, which should encourage us to arrange ophthalmic examinations in accordance with patients’ IS taper, DLI, or vaccination schedules rather than every 3 or 6 months. Certainly, whenever patients complain about painless mucous discharge, tearing, and crusting, they should be seen immediately.

Another finding is that ETCL is more likely to involve the superior tarsal conjunctivae (32 of 35 eyes) than the inferior ones (18 of 35 eyes). Therefore, the careful inspection of all ocular surfaces including the everted upper tarsal conjunctivae should be adopted in routine practice.

According to the 2014 National Institutes of Health Consensus, there are two principal categories of graft-versus-host disease: chronic (cGVHD) and acute (aGVHD), differentiated according to clinical presentations and features [1,29]. aGVHD occurs when the donor stem cells successfully populate into a functional immune system and start to attack the host. The typical target organs of aGVHD include the skin, the gastrointestinal tract, and the liver. The ocular involvement in aGVHD is probably under-recognized in the context of other, more life-threatening conditions. Ocular aGVHD has been reported to be associated with dry eye sensation (often without epitheliopathy), foreign body sensation, (bulbar) conjunctival hyperemia, and, in severe cases, pseudomembranous conjunctivitis [3,18,30,31]. Chronic oGVHD is often diagnosed at a late stage when the ocular irritation has exceeded the patient’s high tolerance threshold. It has been reported to be associated with prominent fibrosis of the conjunctiva, and with ocular surface epitheliopathy, KCS, and a greater risk of corneal perforation in extreme cases [32]. A subset of cGVHD—overlap syndrome—is described as a combination of traditional cGVHD symptoms and one or more aGVHD symptoms [17,32]. Such categorization of GVHD is still dominant in the HSCT and ophthalmology communities. What we have observed in this series supports the more unifying theory: regardless of the length of time following allo-HSCT, oGVHD activity can lead to an erosive conjunctival process. It is a similar process in both early and later stages, triggered by the donor immune system activation, the intensity of which determines the severity of ETCL. IS taper or immunizations typically trigger a milder form of ETCL than that from the immune reconstitution in acute GVHD, and therefore, is much less noticeable. Even when noticed, it is often misdiagnosed as “infectious conjunctivitis” and treated with topical antibiotics. Further understanding of the ETCL process will significantly increase our understanding of oGVHD pathogenesis.

This is the first ever report of such nodular erosion lesions in the literature. The nodules (Figure 1) were different from the fine papillae seen in allergic conjunctivitis, or the clear, fluid-filled follicles seen in viral conjunctivitis. The nodules can appear individually or in clusters with variable sizes from 1 mm to over 5 mm in diameter. In previous studies, a serosanguineous exudate was reported to progress into pseudomembrane [5], but such nodular lesions were never described. Several studies examining pseudomembrane in the setting of oGVHD have demonstrated that donor-derived immune cells, particularly Natural Killer T-cells, are active in infiltrating the host epithelium [6,33,34]. We hypothesize that nodular ETCL may represent the initial sites of donor immune cell infiltration, with the potential to progress into a larger area of deeper involvement—the pseudomembranous erosion.

To avoid iatrogenic trauma, we did not biopsy any lesions because trauma can lead to non-healing defects in such populations and detrimental outcomes [35]. Instead, we performed an anterior segment OCT and found the lesion to be uniform and hypo-reflective without any fibrous internal structure. Future investigation into the histopathology of the nodular lesions is needed to elucidate the pathogenesis.

We have repeatedly observed the formation of CSEF as the nodular or pseudomembranous ETCL resolves (Figure 3). All the tarsal conjunctivae of the 16 eyes were free of fibrosis prior to developing fibrosis at the exact location of ETCL. Therefore, our observation indicates an origin of the CSEF reported previously [18]. We treated the ETCL in all the eyes empirically with topical steroids. Although this is not a controlled study and no comparisons were made between fibrosis formation in other treatment regimens, we hope the early treatment decreased the level of inflammation and helped preserve the conjunctival epithelium and normal eyelid anatomy, therefore decreasing the risk of severe ocular surface complications in the future [16].

## 5. Limitations

Our series is limited by its retrospective nature, as well as the dependence on patients’ recollection, who have all undergone extensive chemotherapy, conditioning, and treatment for other forms of GVHD. Despite an extensive chart review of the patients’ medical records from their clinic encounters with the HSCT providers, not all ocular complaints were documented prior to our encounters. The self-reported timeline of symptoms and events was limited by their memory. Only 13 out of the 19 patients had rigorous follow-ups in the first 6 months. The others were not seen regularly due to various reasons such as hospitalization and long travel distances. It reflects the difficulty of studying this group of patients whose systemic conditions are very challenging to manage.

This study is a retrospective case series with purely observational findings and is reliant on active processes at the time of examination. It is possible that other unobserved aspects of the disease may have been present at times when the patients were not in the clinic, and/or not apparent due to the lack of physical presentation while in the clinic. Additionally, as most patients did not have documented eye exams prior to their allo-HSCT, there were few documented baseline findings to compare to. Future longitudinal observational studies including a pre-HSCT eye exam are warranted to further elucidate the natural history of oGVHD as strongly recommended by the NIH consensus group [16]. Future controlled studies should also be carried out to explore early treatment options and effects.

## 6. Conclusions

We have observed that ETCL can occur shortly after certain immunogenic events in patients with an established diagnosis of oGVHD and those who developed oGVHD soon after, and that ETCL precedes CSEF in situ. Patients at this stage mostly experience thick mucous discharge and morning crusting with less dryness or other ocular discomfort. Our findings present an opportunity to diagnose and investigate oGVHD much earlier in the disease course.

## Figures and Tables

**Table 1:** Patients’ characteristics.

	Male (n = 12)	Female (n = 7)	Total (n = 19)
Age-year (SD)	58.9 (11.7)	62.6 (7.8)	60.3 (10.6)
Ethnicity			
Non-Hispanic Caucasian	10	7	17
Hispanic Caucasian	1	0	1
Hispanic African American	1	0	1
Conditioning Regimen			
Myeloablative	4	2	6
Reduced Intensity	8	5	13
Donor			
Matched Related Donor (Sister)	3	0	3
Matched Related Donor (Brother)	2	0	2
Matched Unrelated Donor	7	7	14
Stem Cell Source			
Peripheral Blood	11	7	18
Bone Marrow	1	0	1

**Table 2:** Patients’ symptom records at erosive tarsal conjunctival lesions (ETCLs) onset and at 6 ± 1 month follow-up.

Symptom	At ETCL Onset (19 Patients)	At 6 ± 1 Month (13 Patients) *
Mucous discharge	16 (84%)	4 (31%)
Heavy morning Crusting	15 (79%)	3 (23%)
Dryness	9 (47%)	10 (77%)
Irritation/FBS	8 (42%)	9 (69%)

* Thirteen patients had follow-up regularly and had visits at 6 +/− 1 month. Among them, 7 had increased corneal fluorescein staining (CFS), 3 had decreased and 1 had unchanged CFS, and 2 had therapeutic scleral lens. ETCL = erosive tarsal conjunctival lesions; FBS = foreign body sensation. Significant percentages are bolded.

**Table 3:** Mean time from hematopoietic stem cell transplantation (HSCT), systemic immune-suppressive (IS) drug taper, vaccination, or donor lymphocyte infusion (DLI) to erosive tarsal conjunctival lesions (ETCL)-related symptom onset.

Patient	Symptom Onset (Months after HSCT)	IS Taper (Months before Symptom Onset)	Vaccination (Months before Symptom Onset)	DLI (Months Before Symptom Onset)
1	5.6	0.5	-	-
2	12.6	4.3	-	0.6
3	5.6	0.6	0.9 (COVID)	-
4	Unclear	Unclear	-	-
5	21.4	4	0.8 (COVID)	-
6	10.5	0.6	0.7 (COVID)	-
7	9	1.2	-	-
8	6.3	0.3	-	-
9	10.5	1	-	-
10	9	0.9	-	-
11	9.6	-	0.8 (Bundle)	-
12	4.4	1.1	-	-
13	9.7	0.5	0.5 (Bundle)	-
14	8	0.2	-	-
15	9.6	4	1 (COVID)	-
16	8.8	1	2	-
17	9.1	2.9	0.1 (Bundle)	-
18	8	2.3	0.3 (COVID)	-
19	9.6	0.6	0.9 (Bundle)	-

HSCT—allogeneic hematopoietic stem cell transplantation; DLI—donor lymphocyte infusion; IS taper—most recent systemic immunosuppressive drug taper; Vaccination—any vaccination received within 3 months prior to ocular symptom onset; Bundle—post-HSCT 9-month vaccination bundle (DTap, 5 Pertussis antigens, Hib, PRP-T, Pneumococcal conjugate PCV20, Polio—IPV, Zoster recombinant); Unclear—patient’s ocular symptom onset timing was not documented.

## Data Availability

The data that support the findings of this study are available upon request from the corresponding author, Z.K.L.
